# Unexpected Benefits in Single Institution Experience With Successful Implementation of a Standardized Perioperative Protocol in Pediatric Thyroidectomy

**DOI:** 10.1097/pq9.0000000000000568

**Published:** 2022-06-14

**Authors:** Kristina Cossen, Matthew T. Santore, Kara K. Prickett, Steven L. Goudy, Kurt F. Heiss, Kanika Shanker, Adina L. Alazraki, Briana C. Patterson

**Affiliations:** From the * Pediatric Endocrinology, Emory University SOM, Children’s Healthcare of Atlanta, Atlanta, GA; † Department of Surgery, Division of Pediatric Surgery, Emory University SOM, Children’s Healthcare of Atlanta, Atlanta, GA; ‡ Department of Otolaryngology-Head and Neck Surgery and Pediatrics, Emory University SOM, Children’s Healthcare of Atlanta, Atlanta, GA; § Division of Pediatric Endocrinology, Goryeb Children’s Hospital, Atlantic Health System, Morristown, New Jersey; ¶ Pediatric Radiology, Emory University SOM, Children’s Healthcare of Atlanta, Atlanta, GA; ∥ Aflac Cancer Center, Emory University SOM, Children’s Healthcare of Atlanta, Atlanta, GA.

## Abstract

**Introduction:**

To illustrate how quality improvement can produce unexpected positive outcomes.

**Methods:**

We compared a retrospective review of perioperative management and outcomes of baseline 122 pediatric total thyroidectomies to 121 subsequent total thyroidectomies managed by an Electronic Medical Record protocol in a large, free-standing children’s healthcare system. Process measures included serum calcium measurement 6−12 hours postoperatively; parathyroid hormone measurement 6 hours postoperatively; preoperative iodine for Graves disease, and postoperative prophylactic calcium carbonate administration. In addition, we completed 4 Plan-Do-Study-Act (PDSA) cycles, focusing on implementation, refinement, usage, education, and postoperative calcitriol administration. The primary outcome included transient hypocalcemia during admission.

**Results:**

All perioperative process measures improved over PDSA cycles. Measurement of postoperative serum calcium increased from 42% at baseline to 100%. Measurement of postoperative PTH increased from 11% to 97%. Preoperative iodine administration for Graves disease surgeries improved from 72% to 94%. Postoperative calcium carbonate administration increased from 36% to 100%. There was a trend toward lower rates of severe hypocalcemia during admission over the subsequent PDSA cycles starting at 11.6% and improving to 3.4%. With the regular review of outcomes, surgical volume consolidated among high-volume providers, associated with a decrease in a permanent hypoparathyroid rate of 20.5% at baseline to 10% by the end of monitoring.

**Conclusions:**

In standardizing care at 1 large pediatric institution, implementing a focused quality improvement project involving the perioperative management of transient hypocalcemia in total thyroidectomy pediatric patients resulted in additional, unanticipated improvements in patient care.

## INTRODUCTION

### Problem Description

Pediatric Graves disease (GD) and differentiated thyroid cancer (DTC) are rare conditions, with an incidence of 0.9:100,000 for GD^1^ and 0.54:100,000 for DTC.^2^ Thyroidectomy is a definitive therapy for patients with GD and DTC. Recent guidelines from the American Thyroid Association (ATA) were published in 2016 and 2015 for DTC and GD, respectively.^3,4^ Given the relative rarity of these conditions, total thyroidectomy is an uncommon pediatric surgery but one that can result in significant morbidity outside of the operation room. Therefore, when possible, ATA guidelines recommend a high-volume surgeon and management by a multidisciplinary team including surgeons, endocrinologists, and anesthesiologists.

### Available Knowledge

Surgical complication rates are higher in children than adults, with even higher complication rates in younger than older children.^3^ In addition, thyroidectomy complication rates are twofold higher when low-volume surgeons, who do not have extensive current experience in this procedure, perform them compared with when performed by high-volume surgeons.^5^ Thyroidectomy is associated with the risk of multiple complications, which include bleeding, injury to the recurrent laryngeal nerve, and hypocalcemia, with or without unintentional parathyroid removal or injury.

Postoperatively, younger children are also at higher risk for transient hypoparathyroidism than adolescents or adults. Therefore, monitoring serial calcium and parathyroid hormone (PTH) levels in the immediate postoperative period is recommended.^3^ This use of perioperative PTH monitoring may decrease morbidity and allow for stratification of patients who would benefit from more intensive monitoring and treatment with calcium and calcitriol.^3,6^ Despite these recommendations, there is an overall lack of consensus on an ideal prophylactic regimen in pediatric patients leading to variability in the postoperative management of hypocalcemia.

In addition, the ATA recommends universal preoperative potassium iodine administration in preparation for pediatric patients with GD.^4^

### Rationale

As there are no specific guidelines for perioperative management in pediatric thyroidectomy, the reasons for developing a standardized order set came out of the need to address the concern that the lack of consistent monitoring and treatment subsequently led to increased hypocalcemia and length of stay. Also, the ATA has clear guidelines for potassium iodine administration for total thyroidectomy due to GD. Still, there might not be a consistent mechanism to ensure patients are prescribed stable iodine due to multispecialty involvement. Therefore, creating an agreement between the general surgeon and endocrinologist for medication management before and after surgery would be essential for care. In addition, developing an Electronic Medical Record (EMR) order set would make ordering appropriate tests and medications simpler and improve multispecialty involvement.

### Specific Aims

This study aims to assess the current practice for the perioperative medical management of thyroidectomy patients in a pediatric hospital system, determine where variability exists that could impact the quality of care, and implement uniform protocols to enhance the consistency and quality of care.

## METHODS

### Context

This quality improvement project occurred within Children’s Healthcare of Atlanta (CHOA), which includes 2 large tertiary care pediatric hospitals with 574 beds. Pediatric otolaryngologists and general pediatric surgeons perform total thyroidectomies at both sites.

The team members who developed the targets and interventions consisted of pediatric endocrinologists, general pediatric surgeons, and pediatric otolaryngologists from the academic hospital. Additionally, the surgical advanced practitioners ensured order sets were implemented during the hospitalization and provided feedback on order set use.

We conducted a 68-month retrospective, cross-sectional study at the 2 hospitals to determine baseline quality of care. During this period, CHOA averaged 21 total thyroidectomies per year. The average number of cases per surgeon annually was 2−3 individual total thyroidectomies. One hundred twenty-two total thyroidectomies performed were reviewed from January 1, 2010, to August 31, 2015. Table 1 shows demographics. Indications for thyroidectomy included GD, DTC, known genetic predisposition to thyroid cancer, large or multinodular goiter, or bilateral benign nodules. For analysis, we grouped the patients as follows: (1) GD, (2) DTC, and (3) others (includes goiter and benign nodular disease and genetic predisposition). We evaluated thyroidectomy admissions if they met the inclusion criteria: total thyroidectomy and patient 2 years and older of age and 21 years and younger of age at the time of the surgery. The exclusion criteria were subtotal thyroidectomy or lobectomy.

**Table 1.** Demographic Characteristics of Pediatric Patients Undergoing Thyroidectomy Baseline and Postquality Initiative

Variables	Baseline Total N = 122	Post Quality Initiative N = 121
Indication, N (%)		
Graves disease	76 (62.3)	52 (43.8)
Thyroid cancer	25 (20.5)	46 (38)
Other	21 (17.2)	22 (18.2)
Sex distribution, N (%)		
Male	28 (23)	37 (30.6)
Female	94 (77)	84 (69.4)
Race, N (%)		
White	55 (45.1)	66 (54.5)
Black	50 (41)	30 (14.8)
Hispanic	11 (9)	11 (9.1)
Asian	5 (4.1)	7 (5.8)
Other	1 (0.8)	6 (5)
Age (y)		
Mean	13.8	14.54
(SD)	(3.82)	(3.41)
Hospital, N (%)		
Hospital A(academic)	58 (47.5)	87 (71.9)
Hospital B	64 (52.5)	34 (28.1)

We defined postoperative hypocalcemia as nadir total serum calcium levels <8.8 mg/dL (2.2 mmol/L) during the hospital admission for thyroidectomy. We further defined mild, moderate, and severe hypocalcemia as levels 8−8.7 mg/dL (2−2.2 mmol/L), 7−7.9 mg/dL (1.75–2 mmol/L), and <7.0 mg/dL (<1.75 mmol/L), respectively. We used these ranges because >8.7 mg/dL is a normal laboratory value at CHOA, and a <7.0 mg/dL serum calcium level typically requires interventions in the form of intravenous calcium for correction.^7,8^ Below normal values not classified as severe hypocalcemia were split equally into mild and moderate groupings. We defined transient hypocalcemia as postoperative hypocalcemia during the hospitalization but without a requirement for supplemental calcium or calcitriol at reassessment 6 months postsurgery. We defined permanent hypocalcemia as hypocalcemia that required calcium or calcitriol supplementation at least 6 months after surgery. Our team followed all cases if on supplementation until their most recent available visit with an endocrinologist as literature supports that patients can show recovery after the 6-month time point and be deemed parathyroid sufficient.^9^

### Interventions

**Fig. 1.**
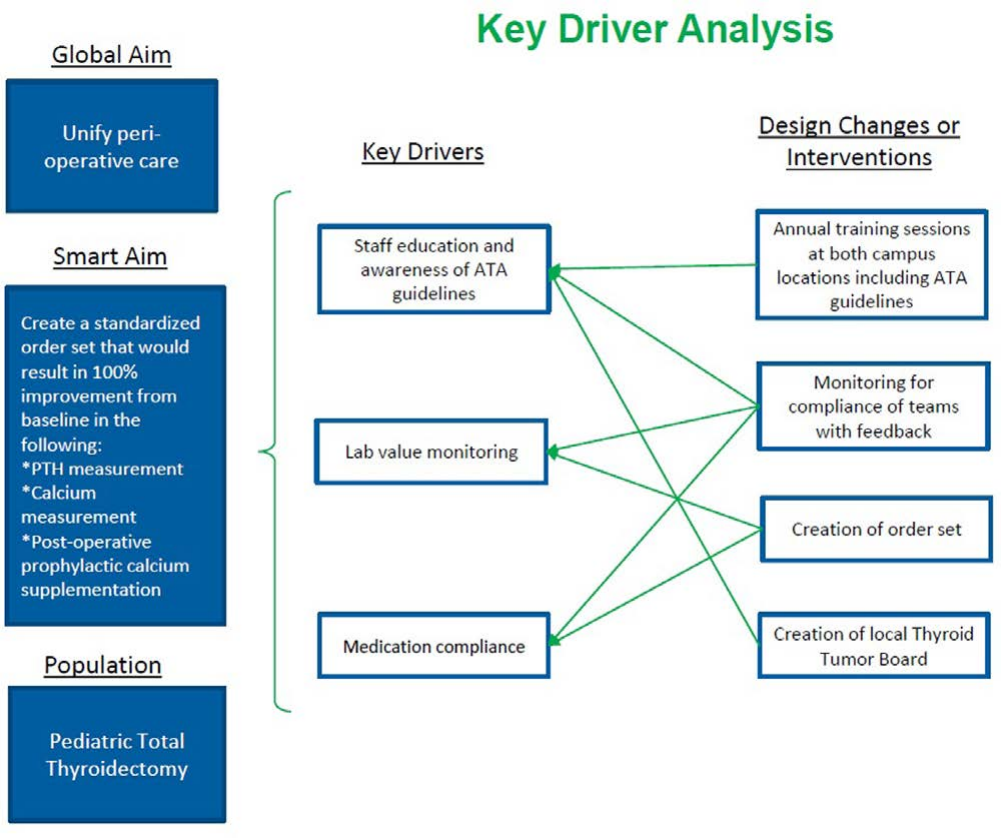
Key driver diagram for inpatient order set initiative

Drawing from the baseline retrospective data, we identified the following targets for patient care improvement (see key driver diagram Fig. 1).

We met with the surgeon groups before the release of the EMR order set to help with awareness:

(1) To standardize the order of a saturated solution of potassium iodide (SSKI) by the surgery team and provide patient instructions on when to begin the medication relative to the surgery date,
(2) To standardize perioperative administration of enteral calcium carbonate, and
(3) To standardize implementing an order set for postoperative monitoring calcium and PTH.

In addition, for our pediatric DTC patients, we initiated a Pediatric Thyroid Tumor Board consisting of endocrinology, otolaryngology, general surgery, pathology, and radiology to discuss the medical and surgical courses.

### Study of Interventions

As part of the first Plan-Do-Study-Act (PDSA) cycle (September 2015–August 2016), the focus was on education of the surgical and endocrine services regarding the ATA guidelines for perioperative care, implementing an EMR protocol to allow for ease of monitoring, and implementing medications when required along with improved communication between hospital services. Surgery teams would be prescribing SSKI in preparation for surgery. Given the increased rates of severe hypocalcemia in patients who did not receive perioperative calcium supplementation, we opted to institute a prophylactic postoperative calcium carbonate regimen with 30 mg/kg/d of elemental calcium. In addition, we implemented inpatient initiation of medications to ensure early introduction in the immediate postoperative period and to improve short-term compliance. We also created recommendations to increase elemental calcium doses based on subsequent calcium labs. Based on ATA guidelines, initiation of calcitriol was based on a PTH level <15 pg/mL,^10,11^ a calcium level <8 mg/dL,^7^ a decrease in calcium >0.5 mg/dL from a previous value,^12^ or a fall in PTH >60% from the previous value^13^ (Table 2). We also included recommendations for medication weaning after discharge from the hospital and biochemical monitoring parameters (Table 2). Finally, we provided audits and feedback on the metrics and outcomes of the changes implemented.

**Table 2.** Quality Initiative Protocol for Total Thyroidectomies at CHOA

Calcium carbonate and calcitriol adjustments in hospital	Initial perioperative care	Labs	Serum calcium levels immediately postoperatively and every 6 h for 24 h then daily until discharge
			Intact PTH level immediately postoperatively and every 6 h for 24 h
		Initial postoperative medications	Calcium carbonate 30 mg/kg/d divided 4 times daily
			Calcitriol 0.5 µg/d*
	Calcium carbonate adjustment	Calcium level 7.7−8.5 mg/dL, increase to 50 mg/kg/d	
		Calcium level 7.0−7.6 mg/dL, increase to 75 mg/kg/d	
		Calcium level <7.0 mg/dL, increase to 100 mg/kg/d	
	Calcitriol Adjustment	Any intact PTH level <15 pg/mL	
		Fall in intact PTH level by >60% relative to prior value	
		Calcium <8 mg/dL	
		Decrease in calcium level of 0.5 mg/dL in comparison to prior value	
Calcium carbonate and calcitriol adjustments after discharge	No in-hospital low calcium and PTH > 15	• Discontinue calcitriol • Calcium wean by decreasing dose frequency by 1 dose per day every 2 d OR reduce from 4 times daily to 2 times daily dosing after 4 d then stop after 4 d on twice daily dosing • Biochemical monitoring in 10−14 d	
	No in-hospital low calcium and PTH < 15	• Calcium wean by decreasing dose frequency by 1 dose per day every 2 d OR reduce from 4 times daily to 2 times daily dosing after 4 d then stop after 4 d on twice daily dosing • Calcitriol wean by 25% of initial dose every 4 d. • Biochemical monitoring in 5−7 d and again in 10−14 d	
	Hypocalcemia in hospital	• Individualized plan for biochemical monitoring and close clinical follow up	

*Addition in PDSA cycle 3.

PDSA cycle 2 (September 2016–August 2017) included a review of current outcome metrics, multiple campus site reviews of protocol, and discussion of order sets in the multidisciplinary meetings with critical stakeholders in the improvement project.

PDSA cycle 3 (September 2017–August 2018) implemented universal postoperative calcitriol to the EMR protocol to decrease rates of significant transient hypocalcemia.^14,15^

PDSA cycle 4 (September 2018–August 2019) included targeted discussions with surgical Advanced Practice Providers (APPs) responsible for order entry to improve the use of order sets.

We included an additional year post PDSA cycles for additional evaluations (Sept 2019 – Aug 2020).

### Analysis

For categorical data, the chi-square test was used. For testing the proportions, a 2-sample proportion test was used. An α level of 0.05 represented statistical significance. A run chart shows the changes with subsequent PDSA cycles.

### Ethical Considerations

This improvement project had no ethical objections. CHOA’s institutional review board determined this quality initiative exempt from institutional review board review.

## RESULTS

The postoperative serum calcium monitoring at baseline showed low rates at the 6–12 hours (41.8%) with a mean nadir serum calcium level of 7.9 mg/dL (SD = 0.73). Initially, our institution measured postoperative PTH levels infrequently (10.2% of patients). However, most of the observed transient hypocalcemia was mild (39.3%) or moderate (37.7%). However, despite inconsistent monitoring, we captured a rate of severe hypocalcemia of 10.7%. We categorized patients into 4 categories for calcium and calcitriol administration: (1) medication before surgery, (2) medication after surgery but before symptomatic hypocalcemia or laboratory value indicative of hypocalcemia, (3) medication in response to hypocalcemia, and (4) no medication during hospitalization. Almost 3-quarters of our baseline cohort patients received some prophylactic calcium either before surgery or after surgery but before the development of hypocalcemia. In addition, almost half of these patients received prophylactic calcitriol. The severe hypocalcemia rates were higher in children who had not received prophylactic calcium supplementation, 6.7% versus 22.6% (see Table 1, Supplemental Digital Content 1, http://links.lww.com/PQ9/A378).

Regarding GD treatment with preoperative SSKI, only 72.4% of patients documented receiving iodine before their procedure.

Throughout the 4 PDSA cycles, there were 91 patients seen for a total thyroidectomy or completion thyroidectomy within our institution and an additional 30 the subsequent year. Table 1 shows the demographics.

**Fig. 2.**
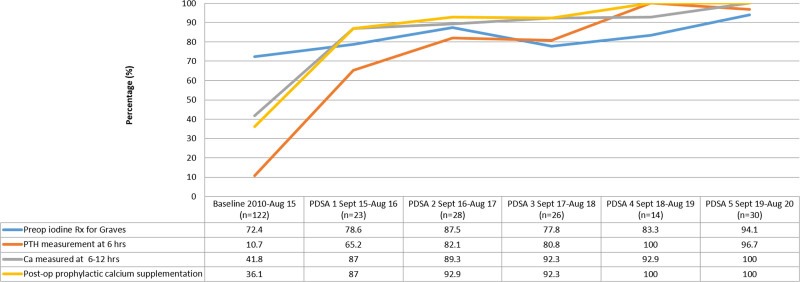
Run chart with preinitiative and postinitiative adherence to order set use and SSKI administration

As demonstrated on the displayed run chart (Fig. 2), we had improvements in all areas of our outcome measures. Following the first PDSA, we provided feedback on the outcome measures and updated education for ongoing care in our multidisciplinary meetings. Rates of prescribing iodine in GD showed a trend toward improvement (72.4% at baseline, 81.8% combining PDSA 1 and 2, *P* = 0.31). Rates of postoperative monitoring of PTH rose from 10.7% to 74.5% combined PDSA 1 and 2 (*P* < 0.01), and serum calcium monitoring rose from 41.8% to 88.2% (*P* < 0.01). Postoperative prophylactic calcium carbonate utilization improved from 36.1% to 90.2% (*P* < 0.01).

**Fig. 3.**
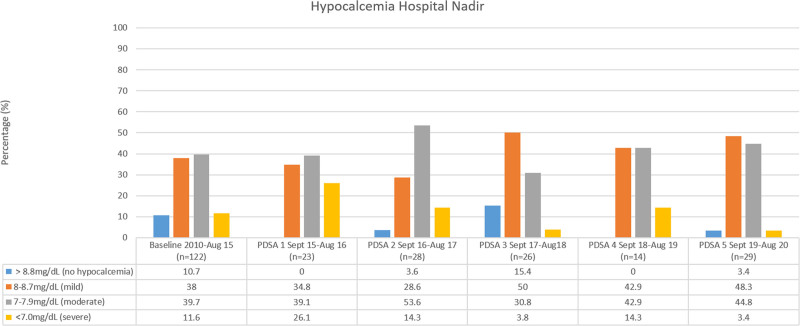
Rates of postoperative hypocalcemia nadir during hospitalization

The rates were unchanged regarding hypocalcemia during hospitalization, but there was a trend toward less severe hypocalcemia (Fig. 3). In addition, hospitalization stays for >1 day remained unchanged, with 39.6% of our patients staying >1 night, compared with a baseline of 40.2%.

## DISCUSSION

This institutional quality improvement initiative reduced variation in care. We aimed to unify perioperative care around total thyroidectomy with an almost entirely achieved 100% uptake of the inpatient order set. This result demonstrates how collaboration across subspecialties improves the monitoring and treatment of transient postoperative hypocalcemia via the universal implementation of EMR order sets. Also, we successfully increased preoperative SSKI use in GD and raised awareness of common issues with hypocalcemia encountered during hospitalization.

Even though the order set utilization improved dramatically, rates of captured hypocalcemia or length of stay were not statistically different from baseline to end of the initiative. When the order set was not used, the reason was usually due to a surgeon who was not commonly performing total thyroidectomies and might not have been aware of this initiative.

This quality initiative is one of the few pediatric examples of prophylactic medications (here postoperative calcium and calcitriol) to reduce immediate postoperative hypocalcemia rates. Vitamin D was not used initially prophylactically or preoperatively for 2 reasons. First, according to NHANES 2001−2004 data, about 9% of the pediatric population was vitamin D deficient and 61% in the insufficient range; preoperative cholecalciferol or ergocalciferol have not been shown to reduce hypocalcemia rates in pediatric thyroidectomy patients.^16,17^ Interestingly, in the article by Tsai et al looking to assess intraoperative PTH and hypocalcemia, they screened their pediatric patients for vitamin D deficiency and treated with D3 replacement or provided 50,000 IU D3 to those with unknown levels. Outcomes showed that initial PTH levels were not related to the vitamin D deficiency status of the patient.^18^ Second, by bypassing the need to use PTH to activate vitamin D and giving calcitriol, 1,25-dihydroxy vitamin D, we had hoped to decrease the transient hypocalcemia seen after total thyroidectomies. However, this result was not observed, likely related to not yet reaching a steady state of calcitriol after a brief hospitalization. We also wanted to document compliance in this quality improvement study, which would be more difficult in the outpatient setting.

However, despite the prophylactic medications, LOS and level of hypocalcemia remain unchanged. Therefore, our team has considered starting vitamin D before surgery to reduce levels of hypocalcemia, leading to increased lengths of stay.

This quality improvement project differs from other pediatric literature in the robust use of oral calcium agents. In a recent review article, intravenous calcium therapy was provided to most pediatric patients for hypocalcemia treatment.^17^ However, our baseline group only had 10 patients (8%) who received intravenous calcium, mostly for tetany and low calcium levels. Only 4 patients (3%) required intravenous calcium during the quality initiative.

What was impressive was the change in culture surrounding these surgeries in the setting of this quality initiative. As a result of the retrospective review of hypocalcemia rates, we noted unacceptable permanent hypoparathyroidism rates. As a result, our surgery teams actively started consolidating toward creating high-volume surgeons with a goal of 1−2 lead surgeons across the system. Figure 1 (Supplemental Digital Content 1, http://links.lww.com/PQ9/A377) displays the differences between our higher volume surgeons and permanent hypoparathyroidism outcomes over the study period. When the 7 surgeons who only had 1 surgery over 10 years were removed, surgeons with >30 procedures were more likely to have lower permanent hypoparathyroidism rates, with a median of 11.7% versus 26.8%. Targeted referrals to high-volume surgeons from our endocrinology teams assisted with this goal. In addition, lead surgeons collaborated with our adult endocrine surgeons to improve the surgical technique for reimplantation of parathyroid glands to help decrease permanent hypoparathyroid rates.

**Fig. 4.**
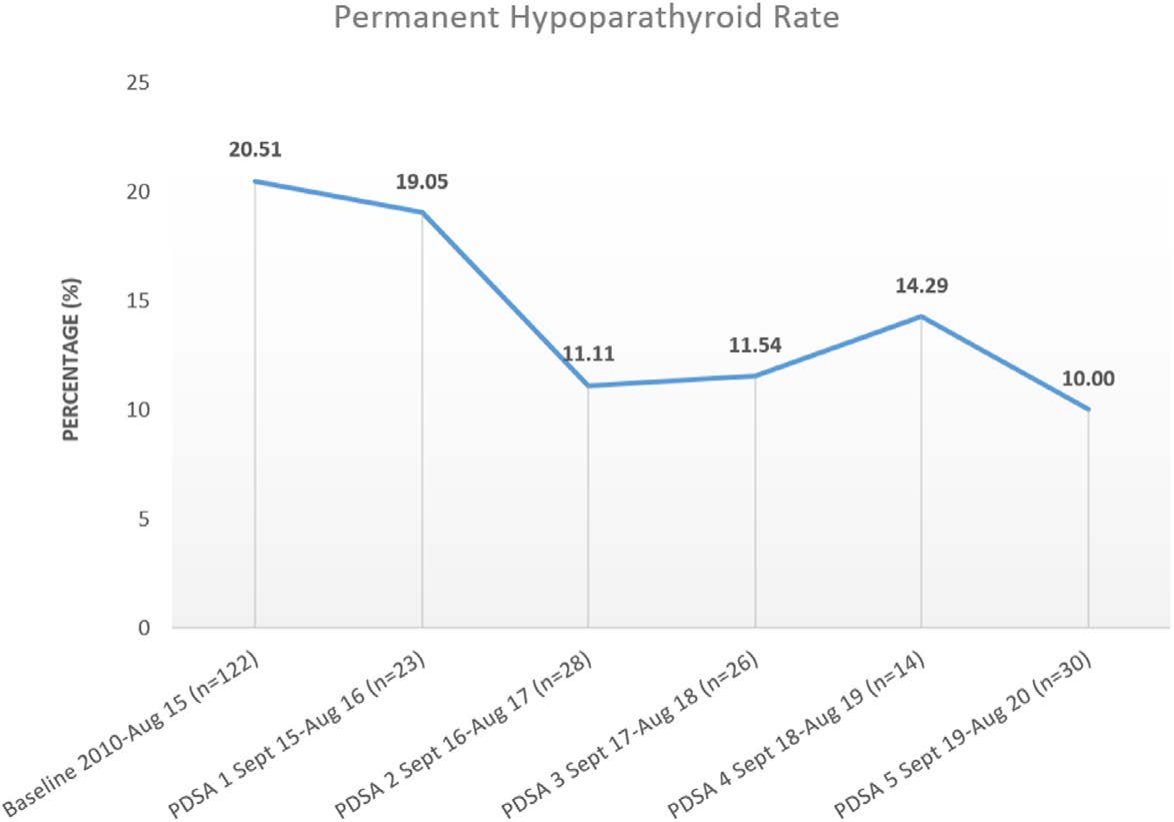
Rates of permanent hypothyroidism throughout the quality initiative

We have seen significant improvements in permanent hypoparathyroidism throughout this project, which was not an anticipated outcome based on preoperative SSKI or perioperative calcium carbonate administration or calcium and PTH monitoring. After completing our initiative, the baseline permanent hypoparathyroidism rate was 20.51%, which improved to 10% (Fig. 4). In reviewing data, the surgical teams made concentrated efforts to create high-volume surgeons within their respective groups and have 2 attending surgeons present for each case, resulting in this improvement in our permanent hypoparathyroidism rate.

Additionally, the Pediatric Thyroid Tumor Board, which meets monthly to discuss active patients, consists of interdisciplinary teams to allow for more rapid and efficient evaluation and communication for patient care. Within this group, expertise and trust have grown and promoted a culture of open communication to benefit error reduction and increase knowledge and technical skills.

### Limitations

One challenge to the universal implementation of the order set and outpatient recommendations was the multiple campus sites with separate endocrine teams; >15 individual surgeons in the Atlanta area available to perform pediatric thyroidectomies in our hospital system, and private endocrinologists providing referrals to the academic center.

Vitamin D baseline status availability was limited and was not included as a quality measure.

This analysis did not aim to collect data on thyroid lobectomy. Thus, the number of thyroid surgeries performed is likely underestimated.

## CONCLUSIONS

Although our protocol for managing transient hypocalcemia did not fully eliminate severe hypocalcemia, we succeeded in standardizing care in a large pediatric healthcare system. There is a continued need for clinical research to generate strong evidence to inform consensus regarding best practices for postoperative care in pediatric thyroidectomy. Without clear evidence-based guidelines for best practice, data from this quality improvement initiative can inform medical decision-making and modify our institutional protocol during our PDSA cycles. However, the most surprising benefit was the increased attention to perioperative care and outcomes on practice patterns in our institution, promoting the consolidation of pediatric total thyroidectomy cases to a smaller number of higher volume surgeons, which ultimately appears to have reduced permanent hypoparathyroidism rates. Although this was not the initial intent of the project, it is an example of an unexpected benefit from collaborative quality improvement efforts between surgeons and endocrinologists.

## DISCLOSURE

The authors have no financial interest to declare in relation to the content of this article.

## ACKNOWLEDGMENTS

Assistance with the study: We would like to thank the surgical nurse practitioners, pediatric and surgical fellows, and residents who helped make this quality improvement project successful.

## Supplementary Material


