# Supplementary material for: Unexpected Benefits in Single Institution Experience With Successful Implementation of a Standardized Perioperative Protocol in Pediatric Thyroidectomy
Source: Pediatr Qual Saf. 2022 Jun 14;7(3):e568. doi: 10.1097/pq9.0000000000000568 (PMC9197358; doi:10.1097/pq9.0000000000000568)
Supplement: Supplementary file 1 [file pqs-7-e568-s001.pdf]

Calcium Administration				Calcitriol Administration			
	Number of patients	Hypocalcemia, n (%)			Number of patients	Hypocalcemia, n (%)	
		Any	Severe			Any	Severe
Pre-operative prophylactic calcium	47	44 93.60%	4 8.50%	Pre-operative prophylactic calcitriol	32	31 96.90%	2 6.20%
Post-operative prophylactic calcium	43	36 83.70%	2 4.70%	Post-operative prophylactic calcitriol	25	22 88%	1 4%
No prophylactic calcium but started calcium for hypocalcemia	16	16 100%	6 37.50%	No prophylactic calcitriol but started calcitriol for hypocalcemia	19	19 100%	8 41.10%
No Calcium Admin	15	11 73.30%	1 6.70%	No Calcitriol Admin	46	35 76.10%	2 4.30%
