# Supplementary figures and images for: Unexpected Benefits in Single Institution Experience With Successful Implementation of a Standardized Perioperative Protocol in Pediatric Thyroidectomy
Source: Pediatr Qual Saf. 2022 Jun 14;7(3):e568. doi: 10.1097/pq9.0000000000000568 (PMC9197358; doi:10.1097/pq9.0000000000000568)

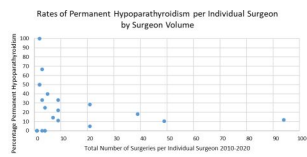

Supplement: Supplementary file 2 [file pqs-7-e568-s002.pdf]
